# Forgetting dynamics for items of different categories

**DOI:** 10.1101/lm.053713.122

**Published:** 2023-02

**Authors:** Antonios Georgiou, Mikhail Katkov, Misha Tsodyks

**Affiliations:** 1 Department of Brain Sciences, Weizmann Institute of Science, Rehovot 76100, Israel; 2 Department of Natural Sciences, Institute for Advanced Study, Princeton, New Jersey 08540, USA

## Abstract

How the dynamic evolution of forgetting changes for different material types is unexplored. By using a common experimental paradigm with stimuli of different types, we were able to directly cross-examine the emerging dynamics and found that even though the presentation sets differ minimally by design, the obtained curves appear to fall on a discrete spectrum. We also show that the resulting curves do not depend on physical time but rather on the number of items shown. All measured curves were compatible with our previously developed mathematical model, hinting to a potential common underlying mechanism of forgetting.

Ever since Ebbinghaus’ seminal work (Ebbinghaus 1964), quantitative measurements of performance have been the staple of human memory studies (e.g., see Kahana 2012). This endeavor is hindered by the sensitivity of memory to multiple factors, such as the material being presented to participants, presentation protocols, age of the participants, etc., that seem to preclude any universal characterization of performance. Following Dudai (2004), we consider memory as a three-staged process consisting of (1) acquisition of information and its encoding, (2) retention in memory over time, and finally (3) recall (we can schematically describe it as *A* → M → Rec). In the following, we assume for simplicity that each of the processes yields a binary result; for example, each presented item is either encoded in long-term memory or not. If a participant is presented with a list of *L* words, some number of them are either missed or not efficiently encoded (i.e., *A* < *L*), those encoded can be erased during the presentation of subsequent words (*M* < *A*), and finally, some number of retained words are recalled (*Rec* < *M*). All three processes could contribute to the unpredictability of memory; however, in our recent publication (Naim et al. 2020), we found that if we can experimentally estimate *M* for a group of participants, their average recall performance can be predicted surprisingly well by a simple phenomenological model of recall, resulting in the universal (i.e., independent of the experimental conditions and type of material presented) formula that relates *Rec* to *M*:

(1) $$<Rec>\approx \sqrt{\frac{3\pi }{2}M}.$$

This implies that under manipulations performed in Naim et al. (2020) (different number of presented items, presentation speed, and item category), most of the sensitivity of recall to experimental conditions is explained by their effect on *M*; that is, on memory acquisition and maintenance during the presentation of the material to be remembered. Indeed, the retention of a presented item in memory requires that (1) it is effectively acquired and stored in memory upon presentation and (2) it is not erased due to interference with subsequent acquisition of new items (forgotten) (see Wixted 2004). In the current study, we focused on the dynamics of forgetting when participants are exposed to a stream of items of a particular type. Following Ebbinghaus (1964), we studied the retention curve [denoted as *R*(*t*) below], which, mathematically, describes the probability that an acquired item still remains in memory after time *t*. Since memory and forgetting are processes that happen in time, exploring their dynamics is of critical importance for probing putative substrate mechanisms of forgetting. In particular, we aim to establish how universal the forgetting dynamics is for different classes of inputs and different presentation conditions.

On the experimental front, even though there have been many studies that explore memory differences between different categories of stimuli such as words, pictures, and sentences (e.g., Jenkins et al. 1967; Shepard 1967; Nelson et al. 1976); within-category differences such as manipulating the level of abstraction within pictures (e.g., Goldstein and Chance 1971; Bellhouse-King and Standing 2007); the conceptual and schematic similarity (Nelson et al. 1976); or the distinctiveness of the material in the format in which it is presented (Ensor et al. 2019), in most cases, memory performance has been addressed as a singular point in time. Rigorous testing of any mathematical model of forgetting as a process, though, requires diverse data as a function of time in accordance with the retention function, which in turn provide a stricter “fitness” criterion. An important question not addressed in previous studies is how generic the shape of the forgetting curve is for different material. We previously demonstrated some degree of universality for the performance of recall by measuring the relation between the number of remembered versus recalled items, acquired in experiments with random lists of words or short sentences (Naim et al. 2020). Could the retention curve exhibit the same universality across stimulus types? The results mentioned above argue against this, but universality could still be observed if the retention curves for different stimulus types are scaled versions of each other; that is, have similar shapes and only differ in absolute values.

Theoretical work has suggested a variety of candidate mechanisms of forgetting with accompanying mathematical models, such as the passive decay of memories (e.g., Kahana and Adler 2017), temporal distinctiveness (e.g., Brown and Lewandowsky 2010), and interference (e.g., Georgiou et al. 2021), to name a few (for an extensive review of decay and interference literature, see Wixted 2004). These theoretical results should then be juxtaposed and compared with experimental findings to assess the validity of their claims. In particular, memory performance varies with the material used in each experiment (see below), and theoretical models should reflect that.

We recently introduced a mathematical model of forgetting based on retroactive interference between acquired memories, which is hypothesized to depend on their relative “valence” measures. More precisely, each memory is represented as a multidimensional vector representing the “strength” (termed valence) of the memory on a number of axes, while a newly acquired memory erases all the previous ones that have a lower valence in all axes (Georgiou et al. 2021). These axes can be different representations of the same item in the semantic space (for example, the word “top” could be the summit, a piece of clothing, etc.) or different perceptual dimensions of the item (visual, auditory, etc.). The inspiration behind having memory items with valences in multiple independent dimensions comes from the observation that at the encoding level, a single item can elicit responses in multiple contextual clusters (Huth et al. 2016). Similarly, at the retrieval level, engram complexes representing a single memory have been reported to be distributed across cortical regions (Roy et al. 2022). If all the valences are assumed to be randomly sampled from an arbitrary distribution, this model can be analytically solved for the retention function. We compared the model predictions with the results of experiments with presentation of streams of randomly chosen common nouns interrupted for recognition trials. The experimentally obtained retention function was shown to be similar to that of the five-dimensional version of our model. As opposed to the recall model of Naim et al. (2020) that had no free parameters, the forgetting model has one parameter that is the dimensionality of the memory valences and hence could potentially account for different shapes of retention function. In the current contribution, we repeated the experiments of Georgiou et al. (2021) with other types of materials, both verbal and visual, to assess the diversity of retention functions across stimulus types and the generality of the retrograde forgetting model of Georgiou et al. (2021).

The model of forgetting proposed in Georgiou et al.(2021) characterizes each presented item as an *n*-dimensional vector, with components sampled from an arbitrary *n*-dimensional distribution. The individual components of this vector represent the valence or, more simply, the memory strength of each particular item in different dimensions. We assume that items are acquired at each time step and committed to memory. Every newly acquired item interacts retroactively with already stored items and erases all of those that are weaker than it element-wise in each dimension (see Fig. 1). This implies that forgetting depends on the number of new acquired items between presentation and test and not on physical time elapsed. Under the simplifying assumption that all components of memory valences are sampled independently from each other, the probability that an item is retained in memory *t* time steps after its acquisition—that is, the value of the retention function *R*_*n*_(*t*)—can be expressed iteratively as

(2) $$R_{n}(t)=\frac{1}{t+1}\sum_{k=0}^{t}R_{n-1}(k),$$

where *n* is the number of dimensions, and *R*_1_(*t*), the retention curve of the one-dimensional model, is simply:

(3) $$R_{1}(t)=\frac{1}{t+1}.$$

**Figure 1.**
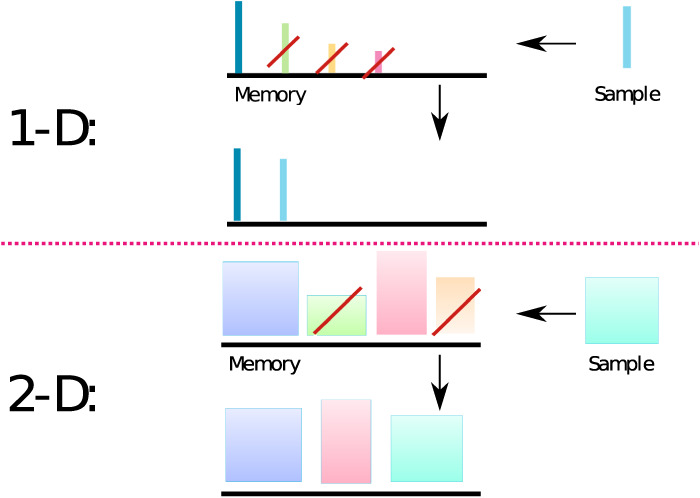
Interference model of forgetting. (*1-D*) For the one-dimensional (1D) version of the model, each item is represented as a colored vertical bar. The height of the bar corresponds to the valence of the item. The *top* row bars *above* the black line represent items that are stored in memory just before the acquisition of a new item, shown at the *right* (sample). All the items that have smaller valence (bar height) than the new item are discarded from memory (crossed by red bar), and the new item is added. The *bottom* row represents the memory content after the new memory is acquired. (*2-D*) Same as in *1-D*, but each memory item has two valence dimensions, represented by the width and height of a rectangle. In this case, all the items that have both valence dimensions smaller than the corresponding dimensions of the new item are discarded.

Equation 3 is trivially obtained by noting that for *n* = 1, the probability that none of the *t* next items will erase the currently acquired one is the same as the chance that out of *t* + 1 independent random numbers sampled from the same probability distribution, the first one is the largest. This chance is clearly given by 1/*t* + 1, since each sample can be the largest one with the same probability. The recursive Equation 2 can be obtained by noting that if we only consider the first dimension, then out of the next *t* presented items, any number of them (*k*) from 0 to *t* can be larger than the present item with equal probability, which is given by 1/(*t* + 1). On the other hand, if there are exactly *k* such items, only they can potentially erase the current one (the rest have smaller valence along the first dimension). The current items can therefore only survive the next *t* steps due to other *n* – 1 dimensions, and the probability for this is then by definition given by *R*_*n* − 1_(*k*). Averaging over all possible values of *k* results in Equation 2.

An equivalent closed-form solution to the retention function can also be found to be

(4) $$R_{n}(t)=\sum_{k=0}^{t}\left(\begin{array}{c} t\\ k \end{array}\right)\frac{{\left(-1\right)}^{k}}{{\left(k+1\right)}^{n}},$$

where $\left(\begin{array}{c} t\\ k \end{array}\right)$ is the binomial coefficient (Katkov et al. 2022). The model yields a family of distinct curves by varying the free integer parameter *n*, with higher *n* leading to a better retention; that is, higher values of retention function (see Fig. 3, below). Recognition experiments’ data for a list of nouns were well described by the model with *n* = 5 (Georgiou et al. 2021).

We expanded the investigation of Georgiou et al. (2021) by conducting similar recognition experiments while changing the presentation material to examine the differences between the retention curves. Participants were recruited online in Amazon's Mechanical Turk platform and were requested to attend to a stream of 200 items at a rate of 1.5 sec per item. At random moments throughout, presentation was paused, and a two-alternative forced-choice (2AFC) recognition task was introduced. Participants were given a choice of two items: one that had appeared before and another one that was not previously shown (see Fig. 2). They were instructed to select the one they remembered seeing (see Georgiou et al. 2021 for more details on methods). The targets of the 2AFC queries were of two kinds. One kind was comprised of the first 10 presented items. These items were inquired at random order and at random times throughout the experiment, yielding 10 responses per participant to 2AFC tasks, with a lag spanning up to 199 items. The results of this kind of query are depicted in Figure 3, where the probability of a correct response (*C*) is plotted versus the number of intervening items between presentation and test, grouped into 10 equidistant bins. As in Georgiou et al. (2021) and many other previous studies, we related the probability of correct response after a certain number *t* of intervening presentations to the probability that the corresponding item is still in memory [i.e., retention function *R*(*t*)] by the equation

(5) $$C=\frac{R+1}{2},$$

which follows from the assumption that if the item is in memory, a participant chooses the correct target at the recognition test; otherwise, the participant chooses an item randomly. The other kind of recognition query was used to select participants who were attentive for the whole duration of the experiment. Another 10 2AFC queries were presented at random times during the experiment—only in this case, the inquired item was always the one before last item (two-back task). This item should still be in working memory, and attentive participants were expected to have 100% accuracy in these tasks. Only the participants that achieved a perfect score for the two-back task were included in the analysis. For all experiments conducted in this study, ethics approval was obtained by the Institutional Review Board of the Weizmann Institute of Science, and each participant accepted an informed consent form before participation.

**Figure 2.**
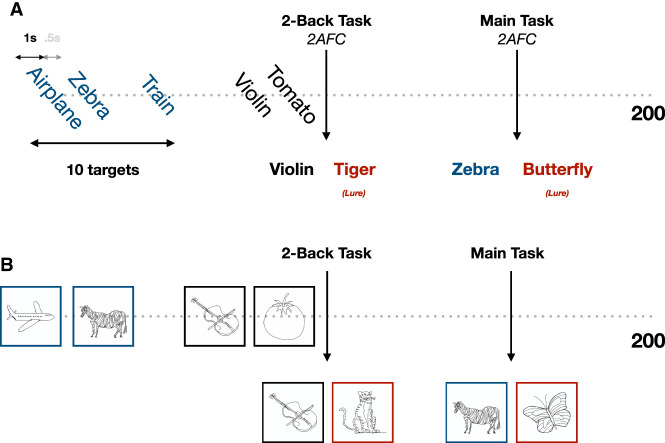
Experimental protocol: Items are presented sequentially for 1 sec followed by 0.5 sec of blank screen. At random points, the presentation is paused, and a two-alternative forced-choice recognition task is shown. The target (stimulus previously shown) comes from one of the first 10 presented items (main task) or the second to last shown item (two-back task), which is used to filter inattentive participants. The distractor (stimulus not previously shown, or “lure”) is shown in red. Stimulus examples of the noun experiment are shown in *A*, and examples of the sketch experiment are shown in *B*.

**Figure 3.**
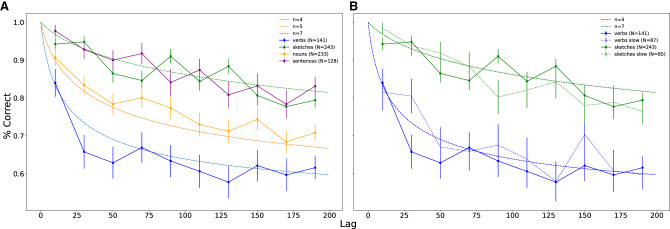
Experimental retention curves for lists of different types and corresponding theoretical curves with different parameter values. (*A*) Fraction of correct recognition versus number of stimuli between presentation and test, grouped into 10 equidistant bins, obtained from a 2AFC memory task on lists of 200 items for different types of material (verbs [blue], nouns [orange], sentences [purple], and sketches [green]) with a presentation speed of 1 sec (where the stimulus is shown) plus a 0.5-sec interitem interval (blank screen). (*B*) Similar to *A* for verbs and sketches with two different presentation speeds. The solid lines represents the results for verbs and sketches as they appear in *A*, while the dotted lines represent the same experiment as in *A* but with a presentation speed of 2 + 0.5 sec. The data obtained from a longer presentation time do not differ substantially from their faster counterparts. In both plots, the dashed lines correspond to theoretical curves produced by our retroactive interference model for different values of the dimensionality parameter *n* (blue: *n* = 4, orange: *n* = 5, and green: *n* = 7). The number of participants contributing to each curve is denoted by the value of *N* in the bracket next to each category.

To examine the difference between verbal and pictorial stimuli, we conducted identical experiments using the same items, only in one case they appeared as sketches and in the other they appeard as words (labels) (see Fig. 2 for some examples). The sketches were simple black and white human-drawn line drawings of different objects (Eitz et al. 2012), while the names of these objects were used in the word experiment. The resulting retention curves of these two experiments can be seen in Figure 3A, where the green curve represents the sketch condition and the orange curve represents the label (word) one. The two conditions give rise to two distinct nonoverlapping curves, with the performance for sketches being consistently higher for every time bin. Comparing these two curves with the curves generated from our model, we see that the results for sketches follow reasonably well the theoretical curve with a dimension of 7, while the labels lie close to the one with the dimension of 5.

A question that arises is whether the observed difference in retention of different stimulus categories is simply the product of verbal versus pictorial stimuli. To address this, the next experiment was conducted using trains of sentences as stimuli. These sentences were small pseudodefinitional, matter-of-fact statements pertaining to a single word (from the pool of words used in Georgiou et al. 2021) such as, “Boats travel on water,” from the seed word “boat.” The resulting curve can be observed again in Figure 3A (purple curve). The general performance is higher than the word condition, as might have been expected, but more interestingly, the curve is almost indistinguishable from the sketch curve, lying around the theoretical curve for *n* = 7.

Following the same train of thought, we introduced another set of stimuli to the same experiment. In our label (words) condition, the list was comprised of nouns, as is typical of recognition experiments of this kind. A minimal modification to this experiment would be to use verbs instead of nouns. The list of verbs was generated by selecting all the verbs from the WordNet database. The verbs that double as nouns were excluded; like, for example, smoke. Then, they were ordered according to frequency based on a variety of online corpora, and the 1000 most frequent were selected. Verbs were previously shown to be remembered worse than nouns when participants were exposed to natural material like phrases or passages (e.g., see Wearing 1970; James 1972; Reynolds and Flagg 1976), but comparisons between lists of verbs and nouns were not performed to the best of our knowledge. Indeed, we found a poorer recognition for verbs, with the resulting retention function quite close to the one predicted by our model with *n* = 4 (see Fig. 3A, blue curve).

Our interpretation of the above results and their comparison with the theoretical model of Georgiou et al. (2021) depends crucially on the assumption that the initial degree of encoding of presented items in long-term memory does not systematically differ for different stimulus categories. In other words, that the initial recognition of items immediately after acquisition, if you disregard the working memory effects, is the same for all stimuli (in other words, extrapolation of the retention curves to *t* = 0 is close to perfect independently of the type of stimulus for participants who pay close attention to all the presented stimuli). An alternative interpretation of the results could be that, for example, verbs are encoded in long-term memory differently than other stimuli such that their immediate recognition is less precise, and this in turn results in a faster rate of subsequent forgetting (e.g., see Wixted 2022). We speculated that in this case, if the presentation time of verbs were increased, the initial encoding would be stronger and the retention curve should move in the direction of the other stimuli. We therefore performed additional experiments in which we increased the presentation time for each verb from 1 to 2 sec. Our results showed, however, that while the relative number of participants with perfect two-back recognition went down (possibly because of the longer overall duration of the experiment), their retention curves did not change significantly apart from one point (see Fig. 3B). For the sake of generality, we performed the same manipulation with sketches and again obtained very similar retention curves for both presentation speeds. We believe that our results point to quite a high degree of universality in forgetting dynamics; namely, when controlled for acquisition (in our case, only considering participants who attend to each stimulus) and stimulus category, retention curves exhibit generic shapes that do not seem to depend on other experimental conditions. We also confirmed our initial assumption that forgetting depends on the number of intervening stimuli rather than on elapsed time. It remains to be seen whether this conclusion will hold for other experimental manipulations not considered in this study, such as the age of the participants.

Taken together with the results of Naim et al. (2020), we conclude that two of the processes involved in memory for random lists of items (namely, retention and recall) exhibit quite a significant degree of universality. The dependence of retention dynamics on the type of the presented stimuli contrasts retention versus recall, which appears to be independent of the item category. One possible explanation for the category-dependent retention is that different stimulus types are processed by a different number of brain areas (e.g., see Hasson et al. 2002). We also note that both these models are deterministic; for example, given the exact values for each presented stimulus, we could predict exactly which one of them will be erased and at which time. How close this is to reality is an open and interesting question, as well as whether extending the model to allow for probabilistic interference, possibly depending on the difference in valences between the items, could provide a better account of the data.

Retention curves could reveal important insights into mechanisms of forgetting and provide a testing ground for theoretical models. In the present study, we have used the recognition experimental paradigm to examine different, but not unrelated, types of material. Interestingly, we see that within the verbal domain, with a difference as minimal as nouns versus verbs, two distinct curves emerge. On the other hand, even between domains (in our case, verbal [sentences] and pictorial [sketches]), the resulting curves are practically indistinguishable. An interesting overarching observation on all the stimulus categories that we considered in this study is that retention curves appear to be either quite distinct or fully overlapping. This observation is consistent with our model of forgetting that has one discrete parameter; namely, the number of dimensions of the memory valence. Moreover, all the data show compatibility with the model for different numbers of dimensions, from four for lists of verbs up to seven for lists of sketches and sentences. We are therefore tempted to speculate that possible retention curves for all types of stimuli form a discrete set of “universality classes” rather than a continuum. More experiments with different categories of items should be performed to either confirm or reject this speculation.

In each experiment conducted in this study, we have looked at retention curves that emerge from presented material coming strictly from a single category. Every presentation train contained only sketches or only nouns, for example. In future studies, there are many categories to explore apart from the narrow selection considered here, such as sounds and natural scenes. Outside of experimental conditions, however, people are bombarded with input comprised of different categories, in different levels of abstraction, and in different modalities simultaneously and sequentially. It would be reasonable to expect that this diverse information would interact and interfere with each other. However, predicting the forms of the resulting retention curves is a nontrivial task. It would be interesting to examine whether these new “complex” curves remain a discrete set or populate the entire retention space.
